# Immunopathogenesis of tuberculosis: cellular mechanisms and immune modulation

**DOI:** 10.1186/s13567-025-01670-1

**Published:** 2025-11-27

**Authors:** Irene Agulló-Ros, Inmaculada Moreno-Iruela, Mercedes Domínguez, José Carlos Gómez-Villamandos, María A. Risalde

**Affiliations:** 1 Departamento de Anatomía y Anatomía Patológica Comparadas y Toxicología, Grupo de Investigación GISAZ, UIC Zoonosis y Enfermedades Emergentes ENZOEM, Universidad de Córdoba, Córdoba, Spain; 2 Unidad de Inmunología Microbiana, Centro Nacional de Microbiología, Instituto de Salud Carlos III, Majadahonda, Madrid, Spain; 3 CIBERINFEC, ISCIII, CIBER de Enfermedades Infecciosas, Instituto de Salud Carlos III, Madrid, Spain

**Keywords:** Cytokines, granuloma, immunopathology, mycobacteria, tuberculosis, vaccines

## Abstract

Tuberculosis (TB) is a worldwide zoonotic disease caused by bacteria members of the *Mycobacterium tuberculosis* complex (MTC), which affects a wide range of domestic and wildlife species, as well as humans. TB is characterized as a chronic pulmonary infection, primarily affecting the lungs and local lymph nodes (LNs), causing significant respiratory and immunosuppression problems. MTC members have the capability to survive in the host by evading the immune system’s killing mechanisms and persisting within macrophages. This chronic antigenic stimulation promotes the formation of a complex, organized tissue structure known as a tuberculous granuloma, which is a defining cellular response to mycobacteria infections, and is composed of a compact aggregate of immune cells, whose functions are modulated by cytokines. The immune response against TB is complex and nowadays is not completely understood; therefore, the study of its immunopathogenesis becomes essential for evaluating immune-mediated response against mycobacterial infections, and consequently, develop strategies to control and eradicate the propagation of this disease in animals and humans. The aim of this work was to review the literature on key cell populations and immunological markers involved in the formation and development of granulomas in the lungs of humans and animals, and to discuss their potential use in evaluating the efficacy of novel vaccine candidates ‒ a tool that could contribute to TB control.

## Introduction

Tuberculosis (TB) is a multi-host worldwide zoonotic disease caused by bacteria members of the *Mycobacterium tuberculosis* complex (MTC) [1], composed, among other species, by *Mycobacterium tuberculosis (M. tuberculosis), M. bovis and M. caprae*. Although these mycobacterial species can infect a broad range of hosts, *M. tuberculosis* is predominantly associated with human infections, whereas *M. bovis* and *M. caprae* are more commonly found in domestic and wild animals [2, 3]. TB is characterized as a chronic pulmonary infection, primarily affecting the lungs and local lymph nodes (LNs) [4], causing significant respiratory and immunosuppression problems [5–7]. Nowadays, TB remains one of the most persistent and challenging-to-control infectious diseases worldwide. This debilitating disease is a cause of morbidity and mortality in livestock, wildlife and humans. Each day, more than 4100 people die from TB, and approximately 30 000 new infections occur globally [1]. Although anti-TB drugs are available, and success rates in humans remain insufficient, aggravated by the growing emergence of multidrug-resistant TB strains [8, 9]. Moreover, medical treatment is not feasible in animals [10]. These factors make vaccination a crucial tool in combating TB, particularly in endemic regions [8, 9], where it has proven valuable in the fight against the disease in humans [8, 9], and badgers [11].

**Figure 1:**
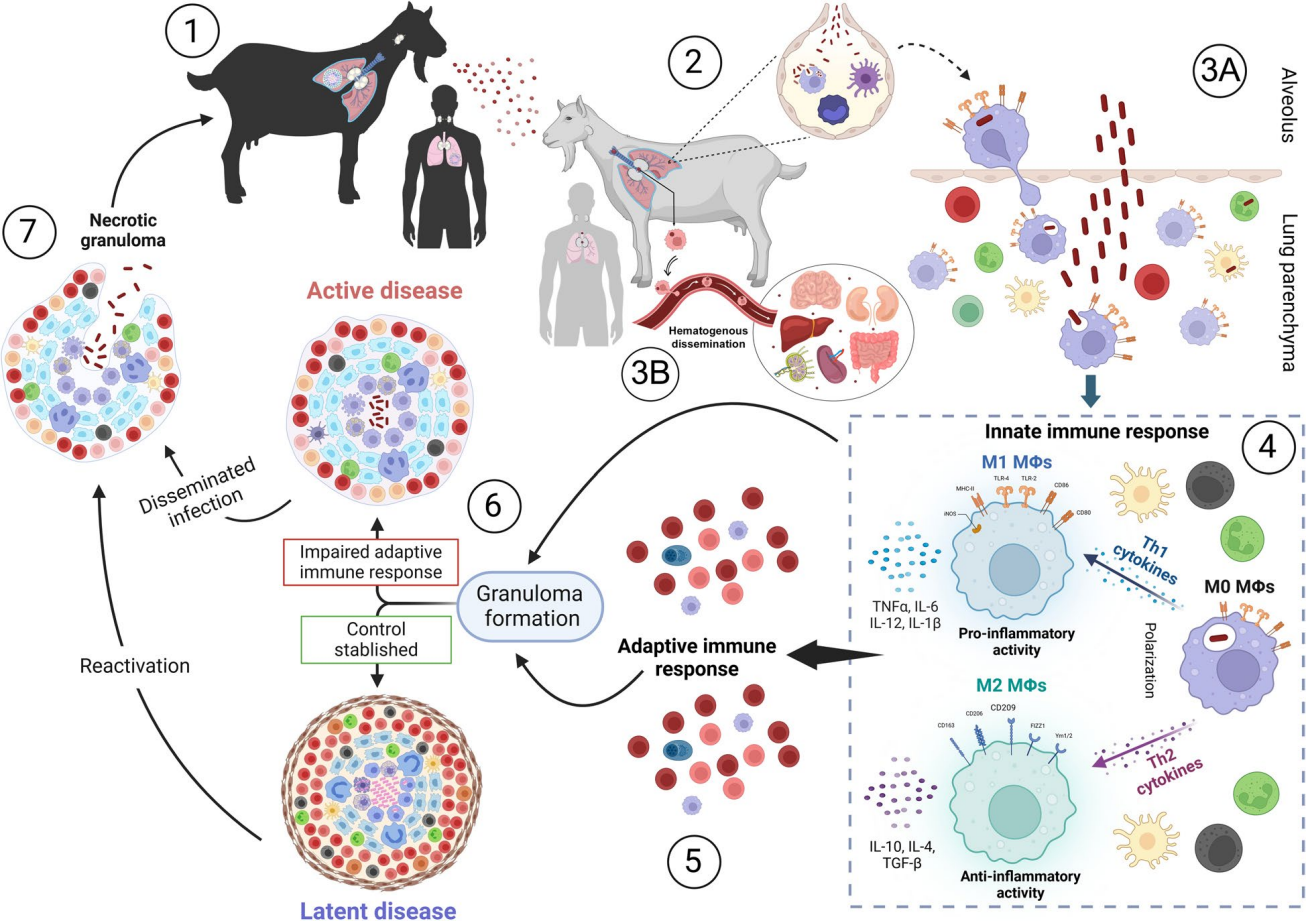
**Schematic representation of tuberculosis (TB) pathogenesis and the immune mechanisms involved in granuloma formation.** The figure illustrates TB pathogenesis, beginning with aerosol transmission of *Mycobacterium tuberculosis* complex (MTC) bacteria during coughing by an infected animal with active disease (1). After inhalation, bacteria reach the alveoli, where they encounter macrophages (MΦs), dendritic cells and monocytes (2). MΦs phagocytose the bacteria, but MTC can evade destruction, replicate within MΦs and migrate into the lung parenchyma (3A). Alternatively, lymph nodes (LNs) can also play a very relevant role in the first encounter with mycobacteria, enabling their potential dissemination through the hematogenous route to other organs and between different LNs (3B). The interaction with mycobacteria triggers the innate immune response, with macrophage polarization into pro-inflammatory (M1) or anti-inflammatory (M2) phenotypes, cytokine release and recruitment of immune cells such as neutrophils and natural killer (NK) cells (4). These innate responses modulate adaptive T- and B-cell activation (5), leading to granuloma formation, which aims to contain the infection (6). In some cases, this results in latent infection, but impaired immunity can cause granulomas to progress to necrotic forms, resulting in active disease and bacterial dissemination (7). Reactivated latent infections or active disease allow MTC to spread via aerosols, perpetuating the transmission cycle.

*Mycobacterium tuberculosis* complex is mainly transmitted via the respiratory route by inhaling infected droplets, while other routes such as ingestion or direct contact are less common [1]. The efficacy of disease progression depends on mycobacteria’s ability to escape the host’s immune response, enabling its survival and replication [12]. However, the immune response to TB is highly complex and remains incompletely understood. Following aerosol inhalation, mycobacteria are recognized by phagocytic cells, such as macrophages (MΦs) and dendritic cells (DCs), through pattern recognition receptors (PRRs) such as toll-like receptors (TLRs). TLR2 and TLR4 play critical roles in mycobacterial uptake and the activation of intracellular signalling pathways, serving as a crucial link between innate and adaptive immune responses [13]. Key players in the innate immune response include MΦs, type 2 pneumocytes, neutrophils and natural killer (NK) cells, which release antimicrobial peptides such as cathelicidins, and cytokines, such as tumour necrosis factor (TNF)α, interleukin (IL)-12 and interferon (IFN)γ (Figure 1) [2, 14]. This cascade of immunological reactions promotes the formation and development of granulomas, which are the classical lesions found in TB and the structures where the interaction between immune responses and mycobacteria will determine the control or progression of the disease (Figure 1) [15].

A deeper understanding of TB pathogenesis, especially the immunological markers that distinguish protective from pathogenic responses, is essential. Although there is currently no fully effective marker [16, 17], identifying robust immune correlates of protection provide valuable insights into the mechanisms underlying protective immunity and disease progression, while also serving as a foundation for evaluating vaccine efficacy. This review is an in-depth exploration of the key immunological markers involved in TB pathogenesis and discusses their potential use in evaluating the efficacy of novel vaccine candidates.

## Materials and methods

This study adhered to the Preferred Reporting Items for Systematic Reviews and Meta-Analyses (PRISMA) recommendations for systematic review reporting [18]. The research question was: what are the key cell populations and immunological markers implicated in TB immunopathogenesis, and how do they contribute to the assessment of vaccine candidates’ efficacy?

The study conducted a systematic search across four electronic databases (SCOPUS, PubMed, Google Scholar, Google search) up until 31 May, 2025. We focused on studies involving TB immunopathogenesis in domestic mammals and humans and collected data using specific key elements. By employing Boolean operators (AND, OR, NOT), we combined search terms and key elements to create search algorithms:

- (Animal models) AND (tuberculosis OR *Mycobacterium tuberculosis* OR *Mycobacterium bovis* OR *Mycobacterium caprae* OR *Mycobacterium tuberculosis* complex) AND (granulomas OR immunopathogenesis OR immune response OR immune markers OR cytokine OR vaccine OR BCG OR immunoprophylaxis).
- (Cattle OR bovine OR goat OR ruminants) AND (tuberculosis OR *Mycobacterium tuberculosis* OR *Mycobacterium bovis* OR *Mycobacterium caprae* OR *Mycobacterium tuberculosis* complex) AND (granulomas OR immunopathogenesis OR immune response OR immune markers OR cytokine OR vaccine OR BCG OR immunoprophylaxis).
- (Mouse OR mice OR guinea pig) AND (tuberculosis OR *Mycobacterium tuberculosis* OR *Mycobacterium bovis* OR *Mycobacterium caprae* OR *Mycobacterium tuberculosis* complex) AND (granulomas OR immunopathogenesis OR immune response OR immune markers OR cytokine OR vaccine OR BCG OR immunoprophylaxis).
- (Pigs OR Minipigs) AND (tuberculosis OR *Mycobacterium tuberculosis* OR *Mycobacterium bovis* OR *Mycobacterium caprae* OR *Mycobacterium tuberculosis* complex) AND (granulomas OR immunopathogenesis OR immune response OR immune markers OR cytokine OR vaccine OR BCG OR immunoprophylaxis).
- (Humans) AND (tuberculosis OR *Mycobacterium tuberculosis* OR *Mycobacterium bovis* OR *Mycobacterium caprae* OR *Mycobacterium tuberculosis* complex) AND (granulomas OR immunopathogenesis OR immune response OR immune markers OR cytokine OR vaccine OR BCG OR immunoprophylaxis).

**Figure 2:**
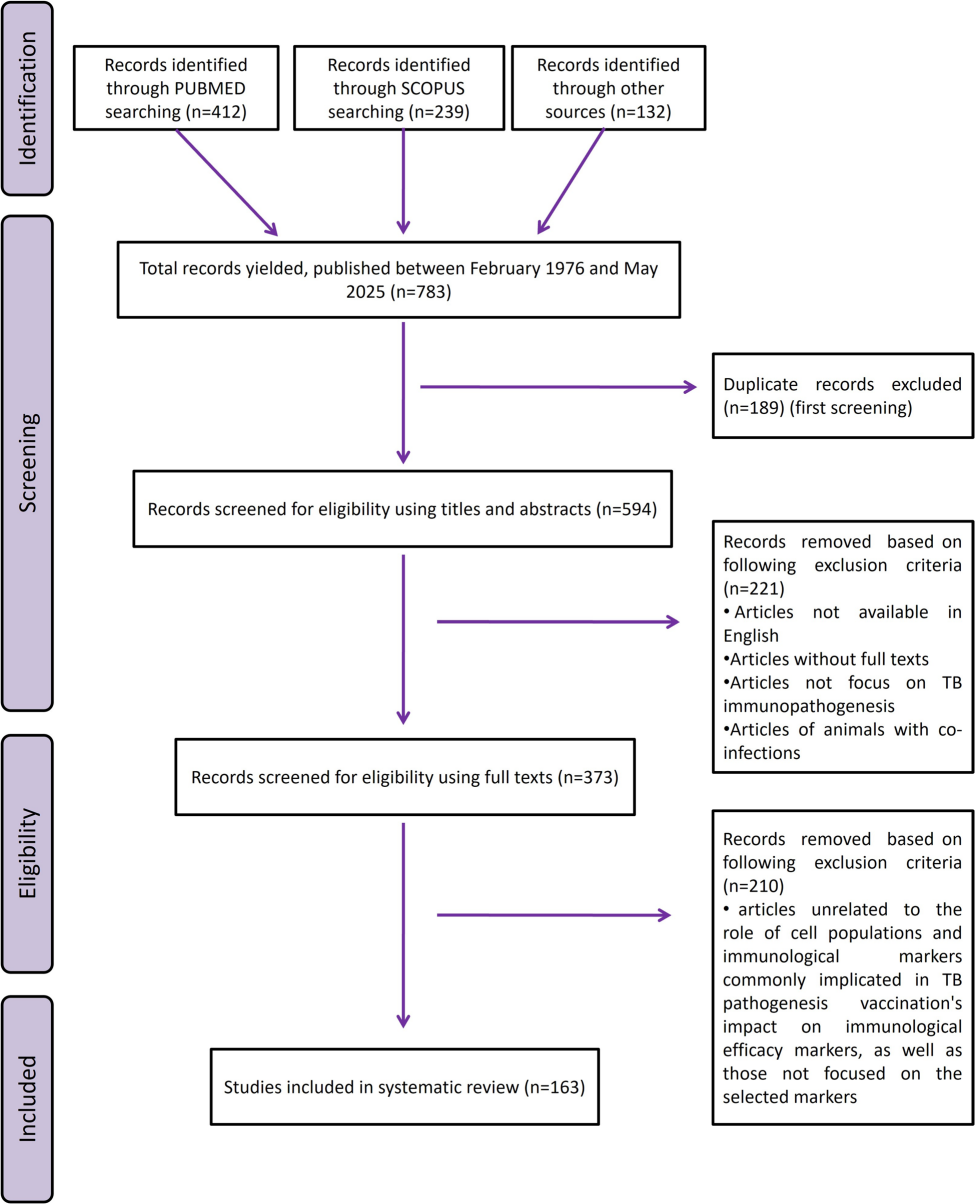
**Scheme followed to carry out the bibliographic review on the basis of PRISMA recommendations.** Source: own elaboration.

The reports obtained for this systematic review underwent three distinct screening phases, as illustrated in Figure 2.

The first screening involved removing duplicate studies when different search engines were combined. The second screening phase included eliminating studies not available in English, articles lacking full texts, redundant articles and those that did not address TB immunopathogenesis. In addition, studies involving co-infections with other pathogens were omitted to eliminate confounding factors that might interfere with the analysis of TB-specific immune responses in which animals were co-infected with other pathogens. In the final screening phase, articles that did not address the role of cell populations and immunological markers commonly implicated in TB pathogenesis were excluded, ensuring the review focused on markers with potential relevance for assessing vaccine efficacy.

## Tuberculous granuloma development and function

The tuberculous granuloma is a lesion that indicates a breakdown in the initial immune defence, where phagocytes fail to eradicate the mycobacteria [19, 20]. This pathological structure is the site of key host–pathogen interactions, determining bacterial replication, killing and latency and subsequently influencing the containment or dissemination of the disease (Figure 1) [21–23]. TB granulomas can present two contrary roles: on the one hand, contain the disease, preventing its spread throughout the organism [23]; on the other hand, they can protect the mycobacteria, allowing it to survive in a latent state for decades in most infected individuals [24]. These individuals can harbour the bacterium for years without exhibiting symptoms, culturable bacilli or clinical signs of disease [25]. However, in cases of immunosuppression, the infection may reactivate, potentially leading to active TB and contributing to its transmission (Figure 1) [25]. Reactivation can occur long after the initial infection, as documented in both humans and *Cynomolgus macaques* infected with *M. tuberculosis* [25, 26]*.* It is estimated that approximately 5–10% of individuals with latent TB will progress to active TB [25, 27]. Reactivation of TB has also been reported in humans infected with *M. bovis*, suggesting it shares dormancy mechanisms with *M. tuberculosis* [28]. Although the bovine immune response to latency antigens is weaker, *M. bovis* harbours the full set of dormancy-related genes and can elicit specific responses in cattle with limited pathology, supporting the potential for latent infection [29–31].

**Figure 3:**
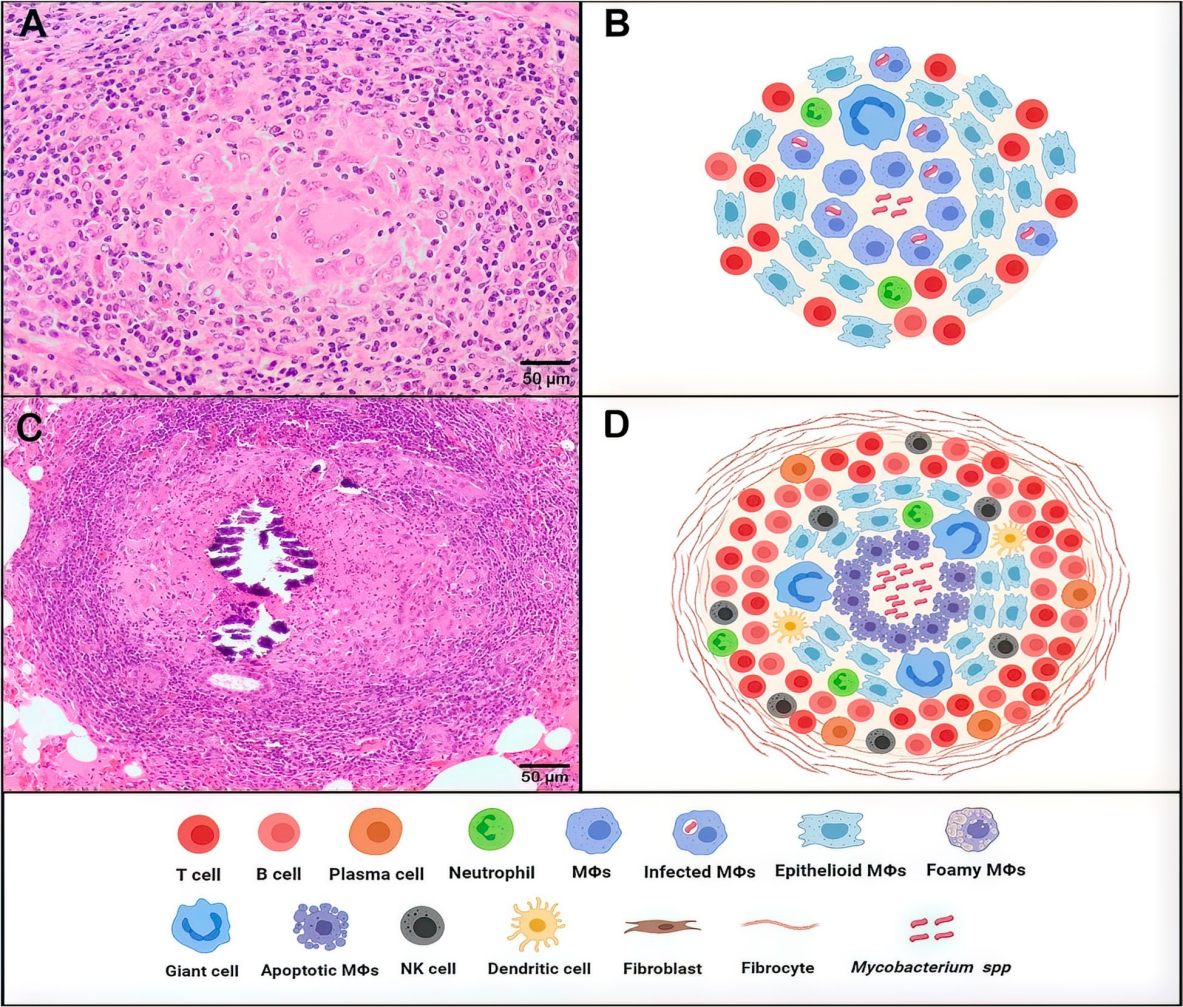
**Schematic and histopathological representation of the evolution of tuberculous granulomas.** The figure illustrates the progression from early-stage granulomas (left), characterized by initial immune cell recruitment and organization, to mature, well-structured granulomas (right) with a defined cellular architecture, necrotic core and fibrotic encapsulation.

**Figure 4:**
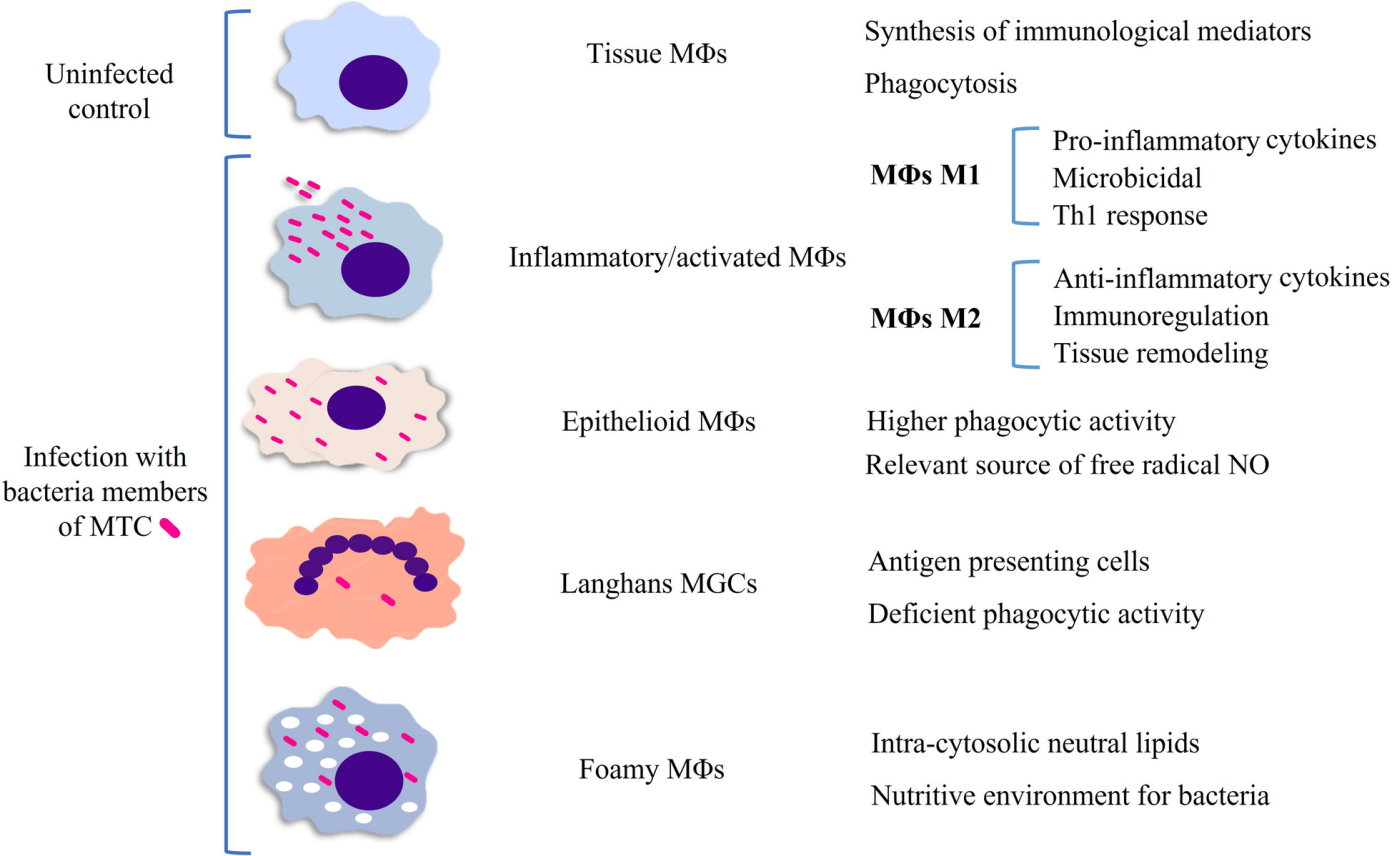
**Phenotypic heterogeneity of macrophages (MΦs) infected with bacteria members of MTC.** Upon mycobacteria infection, MΦs are activated and can evolve into epithelioid MΦs, Langhans multinucleated giant cells (MGCs) and foamy cells (FMs), which present a series of own characteristics and functions.

A tuberculous granuloma begins with the aggregation of MΦs and granulocytes in response to mycobacterial infection, a hallmark of immature granulomas during the early phase of their formation (Figures 3A, B) [32]. Depending on the cytokine environment, MΦs are often classified as classically activated MΦs (CAM) or M1, or alternatively activated MΦs (AAM) or M2 [33, 34] (Figures 1 and 4). M1/M2 polarization of MΦs plays a critical role TB progression or regression, owing to their respective pro-inflammatory (M1) or anti-inflammatory (M2) responses (Figure 4) [35]. M1 MΦs are induced by Th1 cytokines to fight against intracellular pathogens, by secreting pro-inflammatory cytokines (IL-1, IL-12, TNFα) and iNOS with inflammatory effect and bactericidal functions, promoting the Th1-type immune response [24, 36]. Meanwhile, M2 MΦs are induced by Th2 cytokines and exhibit immunoregulatory functions. They mediate pro-healing responses, contribute to tissue remodelling and produce anti-inflammatory cytokines [24, 36]. In the granuloma, MΦs can differentiate into epithelioid cells, characterized by their flattened appearance and elongated nuclei, and may fuse to form multinucleated giant cells (MGCs) with nuclei arranged near the cell periphery (Figure 4) [24]. As the infection progresses, some MΦs accumulate lipids and become foamy MΦs (Figure 4) [37].

Beyond the MΦ population, granuloma maturation is marked by a broader immune response that recruits various cell types, including granulocytes, monocytes, DCs, B and T cells, NK cells and fibroblasts. These cells organize themselves around the MΦ core, contributing to the structural complexity of the mature granuloma (Figure 3C, D) [19, 37]. In the early stages of granuloma formation, mycobacteria actively replicate within MΦs by evading immune clearance through multiple strategies. These include blocking phagosome–lysosome fusion, neutralizing phagosomal pH, detoxifying ROS and inhibiting autophagy and inflammasome activation [25, 38]. Mycobacteria also damage the phagosomal membrane, escape into the cytosol and skew host cell death towards necrosis. A wide array of secreted proteins and lipids disrupt macrophage antimicrobial functions [25]. As the granuloma matures, it becomes a highly organized structure with two distinct pathological features: a necrotic core and a fibrous capsule (Figures 3C, D). The necrotic core, which develops during advanced stages of the granuloma, is indicative of severe pathology driven by dysregulated inflammation and immune failure. This necrosis is associated with a high bacterial load, as the immune system is unable to control mycobacterial replication effectively [20, 37]. In response to this uncontrolled infection, the granuloma develops a thickened outer capsule and often mineralizes through calcium deposition, which represents an attempt to contain bacterial dissemination. The fibrous capsule and calcium accumulation serve as compensatory mechanisms to isolate the infection, but they also highlight the chronic nature and immune dysfunction underlying the disease progression [37]. Therefore, granuloma formation is increasingly recognized as a dynamic and heterogeneous process. Cellular composition, structural organization and immune activity within granulomas can vary widely not only between individuals but also among lesions in the same host. Some granulomas may remain relatively stable, while others evolve toward necrosis and fibrosis.

The variability of TB granulomas is also influenced by host genetic factors [39, 40]. Some individuals exhibit an intrinsic ability to resist natural MTBC infection, effectively eliminating the pathogen without requiring a strong acquired immune response [41]. This natural disease resistance has been documented in humans and several animal species, including mice, rabbits, guinea pigs and cattle [41–43], and is often associated with more efficient macrophage activation, earlier and greater recruitment of IFN-γ–producing T cells to the lungs, improved lymphocyte organization within granulomas and a cytokine profile characterized by higher IFN-γ and lower IL-10 and IL-5 responses, all of which contribute to better bacterial control [42]. Moreover, experimental studies in mice illustrate how host genetics can influence macrophage fate and in shaping granuloma outcomes. Macrophages from C57BL/6, a resistant mouse strain, or from BALB/c mice, preferentially undergo apoptosis upon infection with virulent *M. tuberculosis*, promoting bacterial containment and limiting excessive inflammation associated with necrosis [40, 44, 45]. In contrast, macrophages from susceptible strains, such as C3HeB/Fej, are more prone to necrotic cell death, which may facilitate bacterial survival and dissemination [39]. In addition to genetic factors, age also affects granuloma structure and function [46]. In calves, granulomas tend to contain more bacteria, lack well-organized connective tissue and show fewer immune cells, including epithelioid cells, MGCs and lymphocyte subsets, as well as lower TGF-β levels than in adults [46]. Overall, these features reflect an exaggerated pro-inflammatory response that is less effective at controlling *M. bovis* infection [46].

Overall, the heterogeneity of granulomas highlights the complex interplay between mycobacterial survival strategies, host genetics and age, suggesting that granulomas represent a spectrum of immune outcomes rather than a uniform pathophysiological trajectory [37].

## Role of innate immune recognition and APC co-stimulatory molecules in driving protective responses against mycobacterial infections

The initial interaction between mycobacteria and the host is mediated by components of the innate immune system, particularly through receptors such as TLRs on MΦs and DCs [13, 47, 48]. TLR2 and TLR4 are critical in recognizing mycobacterial components, triggering the activation of MΦs, DCs, NK cells and T lymphocytes, and inducing the secretion of cytokines such as IFNγ and TNFα [13, 47, 48]. In addition, these receptors stimulate the production of antimicrobial peptides, including cathelicidins, which exhibit broad-spectrum antimicrobial activity against various pathogens ‒such as bacteria, fungi, viruses and parasites‒ [49], and play a key role in promoting the migration of inflammatory cells, including neutrophils, monocytes/ MΦs and B lymphocytes [50]. In vitro and in vivo studies have demonstrated that deficiencies in these peptides are associated with reduced intracellular mycobacterial killing and increased bacterial survival within host cells [51–53].

This early immune recognition not only activates the innate immune response but also primes adaptive immunity by enhancing antigen presentation to naive T cells. TLR-2 and TLR-4 are integral to this process, enhancing pro-inflammatory cytokine production, antimicrobial peptide secretion and Th1 polarization, all of which are crucial for an effective immune response [54, 55]. Studies have demonstrated that TLR4 agonists increase CD4^+^ and CD8^+^ T cells in experimental models, reducing mycobacterial burden [56], while TLR4 ^−/−^ mice exhibit impaired immune defence owing to low TNFα expression [47]. Similarly, TLR2 is essential for recognizing mycobacteria and inducing anti-mycobacterial cytokines, including IFNγ and TNFα [13, 47], with TLR2 deficiency linked to poor Th17 differentiation and suboptimal immune responses [57].

In addition to TLRs, co-stimulatory molecules such as CD40 and CD11b, expressed on antigen-presenting cells (APCs) such as DCs, MΦs and B cells, are essential for T-cell activation and Th1 responses [58, 59]. CD40 interacts with its ligand CD40L on activated T cells, leading to APC maturation, upregulation of major histocompatibility complex and production of IL-12, which drives Th1 polarization [58]. CD40-deficient mice, for example, demonstrate increased susceptibility to *M. tuberculosis* infection owing to impaired IL-12 production and deficient IFNγ secretion, leading to uncontrolled bacterial growth and fatal outcomes [58]. CD11b plays a crucial role in adhesion, migration and phagocytosis, and modulates APC cytokine responses and inflammasome activation. It also contributes to the initiation of Th1 responses and enhances host defence against mycobacterial infections by facilitating effective communication between innate and adaptive immune cells [60].

Collectively, these PRRs and co-stimulatory molecules are pivotal in bridging innate and adaptive immune responses. Their roles in antigen presentation, cytokine production and T-cell activation, combined with the antimicrobial function of cathelicidins, highlight their importance in immune defence. In this context, the combined study of these molecules, along with cathelicidins ‒ key components of the innate immune system ‒ provides valuable markers for assessing immune responses, particularly in the development and evaluation of new vaccines against mycobacterial infections [59, 60].

## Current knowledge on the anti-mycobacterial dynamics of MΦs and their immunological mediators in response to tuberculous mycobacteria infection

MΦs, epithelioid cells and MGCs, are the main cell types that compose granulomatous lesions, playing a key role in mycobacterial control [61]. Therefore, studying these populations would be of remarkable interest in evaluating immune response efficacy against TB. The number and distribution of these cells in the granulomas can be evaluated with the CD68 marker for MΦs in general, iNOS for M1 MΦs and CD163 for differentiated M2 MΦs [35]. Higher numbers of M1 MΦs, which secrete pro-inflammatory cytokines such as TNFα, IL-1 and IL-6, and express iNOS, are associated with enhanced host defence against TB [24, 36]. Likewise, the distinct roles of cytokines from MΦs in orchestrating the immune response are critical for controlling mycobacterial infection.

TNFα is a potent pro-inflammatory and pro-immune cytokine, predominantly produced by MΦs, DCs and T and B lymphocytes, that takes part in many activities on both inflammatory and immune responses, including the induction of apoptosis in response to mycobacterial infection [14, 62, 63]. Its presence fosters the expression of a Th1 immune response and plays a significant role in granuloma development and maintenance [3, 64, 65]. TNFα activates MΦs and promotes the expression of chemokines and adhesion molecules, essential for recruiting and retaining inflammatory cells [66]. In bovine, high expression of the TNFα in MΦs infected with *M. bovis* seems to enhance protection against mycobacteria [67]. Mice deficient in TNFα (TNF^−/−^) and infected with *M. tuberculosis* showed a reduced antigen presentation capacity in MΦs and DC [68, 69], failure in granuloma formation, appearing disorganized with diffuse cellular infiltration of neutrophils [66, 69, 70] and higher susceptibility to the infection, resulting in extensive necrotic areas in the lungs and other affected organs [71]. Furthermore, the loss-of-function TNF variant has led to recurrent pulmonary TB in humans [72]. This underscores the indispensable role of TNFα in mediating effective host resistance against MTC.

The IL-1 cytokine family, including IL-1α, IL-1β and IL-1RA, possesses potent pro-inflammatory activity. They are produced by monocytes, MΦs and DCs in response to mycobacterial infection, playing a relevant and dynamic role in TB disease progression [14, 63]. IL-1 participates in the recruitment and activation of phagocytes to the site of infection as well as in the development and maintenance of the granuloma, and consequently, in host defence against TB [24, 73]. In mice infected with *M. tuberculosis*, the absence of this cytokine was related to an extensive pathology in lung, high bacterial load, alteration in the granuloma formation (showing fewer MΦs and lymphocytes and a large number of granulocytes, as well as necrosis) and high mortality rates [73, 74]. Moreover, genetic variations in the *IL-1β* gene in humans have been linked to a higher susceptibility to TB, suggesting that impaired IL-1β production or function may compromise the host’s ability to mount an effective immune response against the infection [74]. However, persistent levels of IL-1 in later stages of the disease can lead to excessive inflammation and tissue damage, potentially impairing the host’s ability to control TB infection [74].

Similarly, IL-6 is produced by myeloid cells in response to TLR stimulation and contributes to the acute phase response alongside IL-1 and TNF-α [75]. It plays a multifaceted role in the immune response and is crucial for CD4^+^ T cell-mediated protection, as IL-6-deficient mice exhibit altered Th1 responses and higher bacterial loads upon infection, underscoring its role in host resistance [76, 77]. Moreover, this cytokine plays an important role in TB pathogenesis by promoting IFNγ secretion, which is essential for generating protective Th1 immune responses, particularly following vaccination with subunit vaccines against *M. tuberculosis* in mice [78, 79]. However, mycobacteria can also manipulate IL-6 production to inhibit type I interferon signalling and suppress IFN-γ responses in uninfected MΦs, thereby facilitating disease progression. Elevated IL-6 levels in the lungs have been associated with TB progression in genetically susceptible mice [80], suggesting a dual role for IL-6: while essential for early immune activation and infection control, excessive or dysregulated IL-6 may contribute to disease pathology [76]. These findings underscore the role of IL-6 as a complex immunomodulator in TB, whose net effect depends on the timing, context and balance of the immune response.

IL-12, a pro-inflammatory cytokine recognized for its strong immune-enhancing effects, is produced by MΦs and DCs [27]. It is a heterodimer composed of two subunits, p35 and p40. The biologically active form, IL-12p70 (p35/p40), promotes the differentiation of naive T cells towards a Th1 phenotype ‒ particularly in the presence of IL-18 ‒ and enhances IFN-γ production in antigen-activated Th1 cells [14, 27, 63, 64, 81]. Its significance in protective immunity against TB has been demonstrated in murine models: exogenous IL-12 treatment improves survival, reduces bacterial burden and enhances granuloma formation [82–84]. Conversely, IL-12 deficiency leads to severe TB in children [85] and impairs granuloma structure and lymphocyte recruitment in mice [84, 86], resulting in uncontrolled mycobacterial proliferation [84, 86]. Importantly, the immunological activity of IL-12 is modulated by the availability and pairing of its subunits. The p40 subunit can form homodimers that act as antagonists of IL-12p70 by competing for receptor binding, thus limiting its protective effects [87]. Furthermore, the p35 subunit is also shared with IL-35, an immunosuppressive cytokine known to promote IL-10 production [88]. This dual usage of p35 and p40 highlights a potential regulatory mechanism whereby the balance between IL-12, IL-35 and p40 homodimers can influence the overall outcome of the immune response against mycobacteria, either promoting protection or contributing to immune evasion [89].

iNOS is an enzyme expressed by MΦs, mature DCs and T cells [90]. Induced by Th1 cytokines, this compound is essential for the production of NO, a toxic molecule generated in MΦs through the oxidation of L-arginine with direct microbicidal activity against a variety of pathogens, including MTC members [33]. Moreover, this enzyme has been positively related with the expression of Th1-type cytokines, such as IFNγ, TNFα and IL-1β, and negatively associated with expression of Th2-type cytokines, such as IL-4, in human TB granulomas [91]. This product and other reactive nitrogen intermediates are essential in protection against mycobacterial infection in animals, such as mice, since it has been observed that NO synthase deficient mice (*NoS*_*2*_^−/−^) infected with *M. tuberculosis* succumb to disease [92]. NO production has been related to MΦ resistance to *M. bovis* infection in cattle [93]. Thus, resistant bovine MΦs exhibit a bacterial load that is two-fold lower and produce higher levels of NO compared with non-resistant MΦs not resistant to infection [94].

## The Th1/Th2 adaptive immune paradigm in TB: central roles of T cells and their immunological mediators in protective immunity

**Figure 5:**
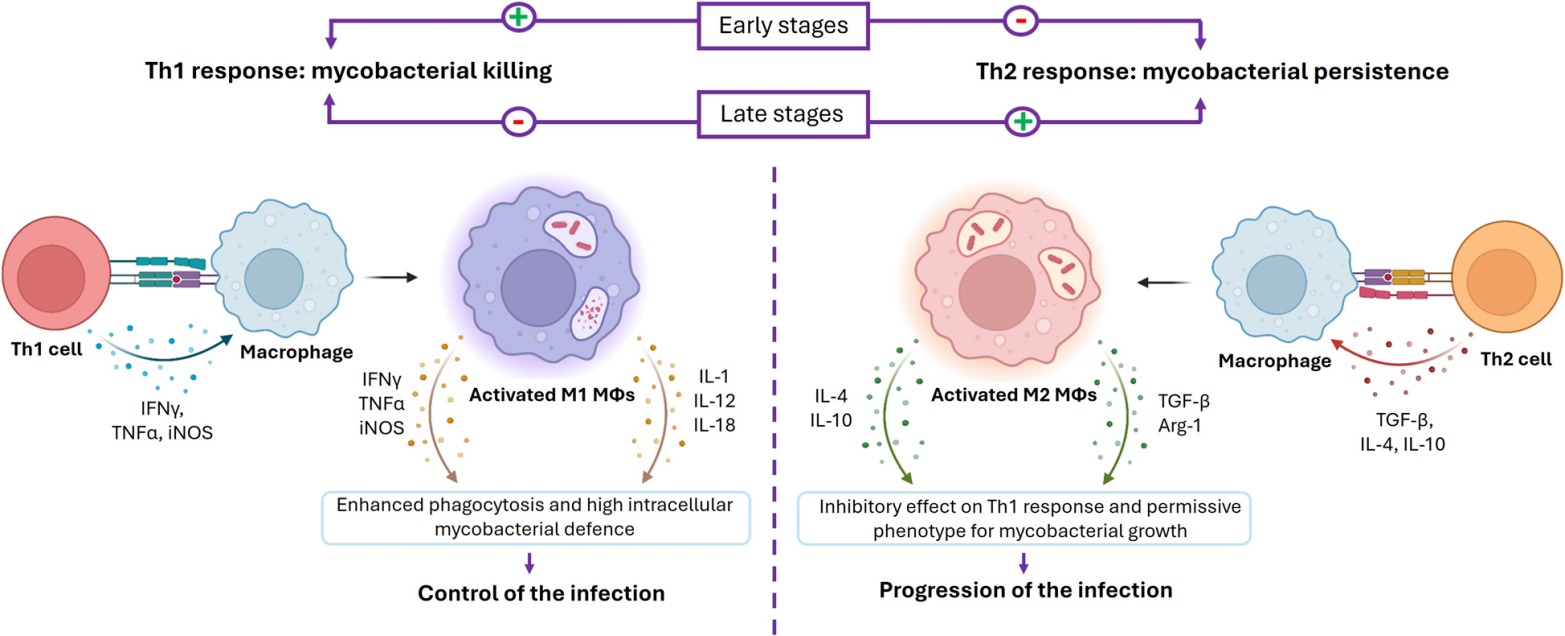
**Immunoregulatory roles of Th1 and Th2 cells in mycobacterial infections.** Th1-type cells exert anti-mycobacterial effects through the production of pro-inflammatory cytokines promoting the polarization of M1 macrophages (MΦs), contributing to improved control in early stages of the disease. Conversely, Th2-type cells secrete anti-inflammatory cytokines that foster M2 MΦs polarization, inhibiting the Th1 response and leading to a productive infection in late stages of the disease.

Adaptive immune responses are central to the host’s defence against mycobacterial infections, with the dynamic interplay between Th1 and Th2 responses critically shaping the disease outcome [95, 96]. Protective immunity against MTC primarily depends on a robust Th1 response, which is characterized by the activation of MΦs, DCs, and the subsequent adaptive T cell-mediated immunity [97, 98]. Th1 responses are crucial for controlling mycobacterial infections, largely through the production of cytokines such as IFNγ and TNFα, which activate M1 MΦs to exert bactericidal functions (Figure 5) [3]. In contrast, the induction of a Th2 response can suppress Th1-mediated immunity, thereby contributing to the progression of TB pathology. Th2-associated cytokines, such as IL-4 and IL-10, polarize MΦs toward an M2 phenotype [34] and downregulate the immune responses needed for effective mycobacterial control, potentially facilitating bacterial persistence within the host (Figure 5) [97, 98].

The dynamic interplay between these Th1 and Th2 responses is critical in determining the outcome of TB infection, with an imbalance towards Th2 contributing to a failure in infection containment [64, 66, 99]. This immune balance is finely regulated by cytokines, which are key signalling molecules that mediate communication between various immune cells [15]. Cytokines play a pivotal role in orchestrating both the innate and adaptive arms of the immune system, modulating the intensity and quality of immune responses against [15]. Understanding how these cytokines shape immune responses provides valuable insights into TB pathogenesis and the response to vaccination.

IFNγ is produced by CD4^+^ and CD8^+^ T cells, NK cells, as well as by MΦs, which are a significant source of IFNγ in the presence of IL-12 [14, 63]. This cytokine, crucial in host defence against intracellular infections, including those caused by MTC members, plays a key role in the development and maturation of granulomas [100]. It induces antimicrobial peptides [51] and a multitude of chemokines [101, 102], facilitating MΦs activation with microbicidal functions [103]. The absence of this cytokine increases susceptibility to virulent *Mycobacteria *spp. infection in both humans and mice, leading to an increase in necrotic granuloma formation [66, 104–106]. However, chronic IFNγ production may contribute to tissue inflammation and reflect a failure to effectively control mycobacteria replication [107]. These findings suggest a dual role for IFNγ, underscoring its essential contribution to protective immunity and granuloma organization, while prolonged expression may reflect sustained immune stimulation in response to unresolved mycobacterial infection [108, 109].

In addition, the expression of CD28 and CD40L (CD40 ligand) has been used as an indicator of T-cell activation in response to MTC infection, as shown in both humans and mice [32, 110]. CD28 plays an essential role for initiating and activating T-cell responses through recognition of various ligands expressed on the surface of APCs [111, 112]. It promotes the differentiation towards effector T cells, clonal expansion, magnitude and duration of T-cell responses, and the production of cytokines such as IL-2 and IFNγ [111, 112], preventing the induction of anergy and favouring the formation of germinal centres [113]. IL-2 promotes the proliferation and maturation of B and T lymphocytes, as well as NK cell cytolytic activity [14, 63, 114]. It triggers an immune response in target cells through a high-affinity receptor (IL-2R) comprising alpha, beta and gamma subunits [115]. Initially, IL-2 interacts with the alpha subunit (CD25), generating a conformational change and increasing its affinity for the remaining subunits [116]. It favours T-cell activation as well TNFα and IFNγ production, showing its role in cellular immunity and the development of granulomas in mycobacterial infection [117]. TB-specific antigens stimulate the release of IL-2 by circulating cells [114]. Moreover, patients with active TB have shown significantly higher serum levels of IL-2Rα compared with those with latent *M. tuberculosis* infection [116]. These findings suggest that the study of IL-2 and its receptor could be a potential biomarker for TB diagnosis [116].

Other authors have reported a notable reduction in activated T cells and quantitative disparities in the cellular composition of the granuloma in infected mice lacking CD28 [32]. Likewise, CD40L is necessary for the generation of *M. tuberculosis*-specific lung Th17 responses for the sustained activation of Th1 in the control of mycobacterial replication at the site of local inflammation [32, 59]. Moreover, it has been reported that the ability to control bacterial growth is notably compromised in mice deficient in CD40L [32]. Therefore, elevated levels of CD28 and CD40L expression could be positive correlated with effector T cells and with IFNγ and IL-17 production [110, 118]. IL-17 plays a critical role in host defence against mycobacteria [64, 66, 119]. It activates MΦs, regulates their antimicrobial mechanisms and facilitates the recruitment of effector cells including neutrophils, contributing to granuloma formation, as shown in murine and bovine models [64, 66]. IL-17 knockout mice infected with *M. bovis* show impaired granuloma development, and elevated IL-17 levels have been linked to animals with TB lesions, suggesting its potential as a TB prognostic biomarker [66].

Another subset of lymphocytes is the regulator T cells (Tregs), which are characterized by the expression of CD4^+^ CD25^+^, the α-chain of the IL-2 receptor [120]. CD25 is highly expressed in these cells, but it is also present in activated T lymphocytes. Consequently, it is commonly employed in conjunction with other markers, such as CD127 or CD39 [121]. These cells possess immunoregulatory properties, as evidenced by their ability to downregulate the activation and proliferation of CD4^+^(CD25^–^) and CD8^+^ T cells through the inhibition of IL-2 transcription [122]. Depletion of Tregs in humans and mice infected with *M. tuberculosis* has been linked to an increase in IFNγ production by CD4 T cells and a decrease in IL-4 production [122]. Moreover, elevated levels of anti-inflammatory cytokines TGF-β, IL-10 and IL-4 are associated with the suppression of T-cell responses and a decreased bactericidal function in MΦs [121], ultimately contributing to increased pathology [99, 123, 124].

TGF-β, mainly produced by M2 MΦs and regulatory T cells, it is a potent stimulator of collagen production and plays a relevant role in the formation of fibrous capsules in advanced-stages granulomas [3, 125]. The absence of TGF-β results in organ failure and death in MTC-infected mice, characterized by extensive areas of necrosis and a multifocal mixed inflammatory cell response, accompanied by wasting syndrome [126, 127]. However, it has also been demonstrated that TGF-β suppresses T-cell responses and reduces MΦs bactericidal function by decreasing the release of TNFα, IL-1, IL-12, IFNγ and reactive oxygen and nitrogen intermediates [97, 128].

IL-10 produced by regulatory T cells, M2 MΦs and B cells, exerts a negative regulatory function on the Th1 response against TB [14, 63]. It induces the polarization of MΦs towards the M2 type and leads to the deactivation of M1 MΦs, thereby inhibiting their microbicidal functions and interfering with APCs’ function [3, 24]. Furthermore, IL-10 modulates the synthesis of pro-inflammatory cytokines [82]. It has been reported that this cytokine acts downregulating different protective host effector mechanisms, such as T-cell proliferation and IFNγ release [99]. Moreover, this cytokine indirectly disrupts the production of reactive nitrogen and oxygen species by suppressing MΦs activation [23, 129]. Thus, it has been demonstrated in humans [130], mice [131] and cattle [99] that elevated expression of IL-10 is linked with a higher susceptibility to mycobacterial infection and increased pathology, while its inhibition favours stronger Th1 responses against MTC [99, 130, 131]. Conversely, studies have shown that IL-10^−/−^ mice infected with *M. tuberculosis* exhibited an increase in CD4^+^ and CD8^+^ T-cell numbers along with enhanced IFNγ expression [23]. For these reasons, the IFNγ/IL-10 ratio is used as an index to determine the disease severity in TB-infected humans and cattle [99, 123].

IL-4 is a pleiotropic cytokine mainly secreted by Th2 lymphocytes and activated mast cells that promotes humoral immunity and drives Th2 cell differentiation [14, 63]. It exerts anti-inflammatory effects by antagonizing IFN-γ-mediated responses, such as M1 MΦs activation and cell-mediated immunity, and by suppressing the development of Th1 and Th17 lymphocytes [114]. It has been proposed that IL-4 has a remarkable role in TB pathogenesis, owing to the downregulation of MΦs, iNOS and TLR-2 [114]. Likewise, high expression of this cytokine coupled with low levels of IFNγ, has been associated to an advanced and progressive TB pathology in humans, increasing the risk of MTC transmission and promoting the conversion from TB latent to active [114]. Moreover, infected IL-4 KO mice exhibited reduced mycobacterial burdens in affected organs, indicating an enhanced level of host resistance to TB [132].

## Unveiling B lymphocytes’ hidden strengths in TB defence

The pivotal role of T lymphocytes in anti-TB defence is well established [133, 134]; however, the specific contribution of B lymphocytes to host defence against mycobacteria remains incompletely understood [135]. In recent years, interest in the role of B cells in TB has increased, with emerging evidence suggesting that B cells modulate T-cell responses, influencing disease progression and outcomes. B cells modulate T-cell activity through antigen presentation, secretion of cytokines such as IL-10 and IL-6, regulate inflammation and impact bacterial load within TB lesions [135, 136]. Indeed, prior evidence suggests that B cells might serve to mitigate the damage resulting from the host response [137], and B cell depletion leads to increased susceptibility to TB in mice [138] and a greater bacterial burden in lung lesions in non-human primates [135]. Notably, innate B cells in human TB have been shown to support a Th1-type immune response through the secretion of IL-12, IFNγ and TNFα [139, 140]. Furthermore, studies in both domestic and wild ruminants infected with *M. bovis* demonstrate a marked increase in B cell (CD79a^+^) numbers during granuloma formation, positioning them as a substantial cellular component within these structures [108, 140–143].

Beyond their regulatory functions, B cells can differentiate into plasma cells that produce antibodies against mycobacterial antigens. Although the protective role of these antibodies remains under debate, growing evidence points to a dual function of B cells in TB. On one hand, humoral responses – particularly those developed within inducible bronchus-associated lymphoid tissue (iBALT) – have been associated with enhanced control of *Mycobacterium tuberculosis* in both humans and animal models [144, 145]. iBALT structures serve as local sites for germinal centre reactions, supporting the maturation of high-affinity B cells and the generation of long-lived plasma cells [146]. On the other hand, excessive or dysregulated B cell responses have been linked to increased pathology [147]. These findings underscore the complex and context-dependent role of B cells in TB, highlighting their potential association with both protection and disease progression.

## Evaluating immunological signatures to assess the protection of new TB vaccine candidates

Vaccination remains one of the most cost-effective strategies for combating infectious diseases, including TB [148]. Epidemiological models suggest that the introduction of novel TB vaccines could prevent 65.5 million cases and 7.9 million deaths in humans by 2050 [149].

The attenuated strain of *M. bovis* Bacillus Calmette–Guérin (BCG), the only commercially available TB vaccine, aims to establish long-term immunological memory in humans and animals not involved in eradication programs [16, 150]. This enables an adequate immune response upon encountering the active pathogen, thereby enhancing host protection [16, 151]. BCG has proven to offer good protection against disseminated TB and meningeal TB in children [16] and is effective in reducing the quantity and severity of lesions, as well as the bacterial load in different animal species under experimental conditions (reviewed in 11). However, BCG also presents several limitations, including a variable efficacy range (0–80%) [17], its failure to provide adequate protective immunity against adult TB in humans [152, 153], an inability to act against latent TB infection [17], local and systematic adverse reactions [154], interference with diagnostic tests in animals [9] and a potential risk of environmental contamination owing to faecal excretion when administered orally [155].Therefore, the development of novel vaccines that can address these disadvantages is essential to achieving the goal of global TB control [17, 156]. However, developing and evaluating effective TB vaccines is challenging owing to the absence of clearly defined immune correlates of protection [157, 158]. Identifying these correlates is essential for evaluating vaccine-induced immunity and for guiding future vaccine development.

The immunological signatures presented in this systematic review have been used as indicators of an improved or non-improved immune response following vaccination against various vaccine candidates, highlighting their importance as key indicators for assessing vaccine efficacy. In this context, it has been reported higher expression levels of TNFα and iNOS and lower bacterial load in the MΦs across all granuloma stages in BCG-vaccinated cattle compared with non-vaccinated animals, suggesting that vaccination contributes to reducing bacterial counts [108]. Similarly, BCG immunization in humans led to enhanced IL-1β production when circulating mononuclear cells were infected with *M. tuberculosis* [159]. This enhanced response may potentially contribute to improved clinical outcomes during infection and could even confer protection against other diseases [160].

DNA vaccines can encode IL-12, inducing cell proliferation, Th1 polarization and protection against MTC [86, 161]. Similarly, a study in vaccinated infants demonstrated an association between IFN-γ-producing BCG-specific T cells and a reduced risk of developing TB disease over the subsequent 3 years [157]. Moreover, an increased number of IFNγ-secreting T cells has been detected in the local tissues of BCG-vaccinated cattle, which was associated with reduced granulomatous lesions and lower mycobacterial burden [108]. Similarly, in BCG-vaccinated mice, stimulation with CD40 agonists and Ag85B-loaded dendritic cells induced strong IFNγ and IL-17 responses in the lungs [118]. Protein-adjuvanted subunit vaccines have also demonstrated the ability to induce robust CD4^+^ T cell IFN-γ, TNF-α, IL-2 and IL-17 in both the lung and spleen and have provided protection against *M. tuberculosis* infection in mice [158]. Nevertheless, higher concentrations of pro-inflammatory mediators are not always associated with improved control of mycobacterial infection [46]. Studies in naturally infected calves have shown that an exacerbated response may reflect reduced microbicidal capacity and increased permissiveness to dissemination [46]. Thus, excessive cytokine production should be interpreted with caution, as it might represent a poor correlate of vaccination, since the effectiveness of the response likely depends not only on its magnitude but also on a balanced interplay with regulatory mediators that ensures bacterial control while limiting tissue damage.

In contrast to the study of pro-inflammatory responses, the study of Tregs and anti-inflammatory cytokines TGF-β, IL-10 and IL-4 could be valuable in identifying markers of vaccine non-efficacy. In this context, a decreased count of Tregs has been correlated with enhanced vaccine protection (BCG/DNA-hsp 65 and BCG/CFP-CpG) in the lungs of *M. tuberculosis*-infected mice [162]. This association has been attributed to the positive correlation observed between Tregs and the colony-forming unit (CFU) counts, with a lower number of Tregs cells being associated to a lower CFU counts [163]. In addition, early neutralization of IL-6 in immunized mice with a subunit *M. tuberculosis* vaccine was shown to decrease CD4^+^ T-cell proliferation and IFNγ release, while increasing IL-4 responses [78]. Furthermore, studies in rats and cattle vaccinated with BCG reported low or undetectable levels of IL-4 and TGF-β in lung [132] and LNs [101, 108], respectively. These findings suggest that vaccination promotes a predominant Th1 immune response, thereby enhancing host defence against MTC [129]*.*

On the other hand, higher B cell numbers have been observed in advanced granulomas of BCG-vaccinated calves compared with unvaccinated, *M. bovis*-infected animals. Although BCG vaccination did not prevent infection, it significantly reduced the number of both gross and microscopic lesions in the lungs and LNs, suggesting a potential association between B cells and modulation of disease progression [108]. These findings point to the possible involvement of B cells in shaping the immune response and warrant further investigation into their role as markers of vaccine-induced modulation of pathology.

## Conclusions

Our research has evaluated the current understanding of immune responses in mycobacterial infections, with a focus on mechanisms that reduce pathology and combat disease. A comprehensive review of the current literature, particularly from the host immune perspective, guided the objectives of this study. This review explored key immune cell populations and immunological markers involved in defending the host against mycobacteria and in the formation and development of granulomas, the principal lesion in TB. Granulomas are dynamic sites of host–pathogen interaction, where immune responses determine bacterial containment or disease progression. Innate immune components such as TLR2, TLR4, CD40, CD40L, CD11b and CD28 on antigen-presenting cells are essential for initiating early immune responses and activating adaptive immunity after MTC infection. Considering this, CD4^+^ T cells, along with IFNγ, IL-2 and IL-17, were identified as pivotal markers of a Th1-dominant response, driving M1 MΦs within granulomas to produce iNOS, TNFα, IL-1 and IL-12, crucial for controlling mycobacterial infection and limiting lesion severity. In contrast, M2 MΦs, which produce anti-inflammatory cytokines such as IL-4, IL-10 and TGF-β, are associated with poorly formed or necrotic granulomas. While a strong Th1 response is central to protective immunity, growing evidence underscores the importance of immune cell subsets, innate immune components, cytokines and mechanisms beyond the Th1 paradigm. We confirmed the complexity of mycobacteria infections and highlighted the ongoing challenges in investigating the pathogenesis and immunoprophylaxis of TB. A key finding was the modulation of the host immune response by mycobacteria, an area often overlooked, particularly during the early stages of infection. This insight provides a foundation for future research and the development of more effective immune-based strategies that can provide broader protection across different populations.

## Data Availability

No datasets were generated or analysed during the current study.
